# What May Be Really Learned from Microscopic Examinations of Urine

**Published:** 1873-06

**Authors:** James Tyson

**Affiliations:** Lecturer on Microscopy and Urinary Chemistry in the University of Pennsylvania


*What may be Really Learned from Microscopic Examinations of Urine.* By JAMES TYSON, M.D., Lecturer on Microscopy and Urinary Chemistry in the University of Pennsylvania.

Few subjects are more imperfectly understood by the mass of general practitioners than that of Urinary Microscopy. Many physicians think that if a specimen of urine is handed to a microscopist for examination, the latter must be able to give such copious and precise information as will unravel all the mysteries of the case, and furnish the key to a speedily successful treatment, or else the instrument is condemned as an expensive luxury, which, if not useless is scarcely of sufficient utility to justify the outlay necessary to procure it. It is indeed true that in a large proportion of instances the information furnished by a microscopic examination of the urine is limited, and that in a smaller number of cases its results are entirely negative.

It is in consequence of the fact that many instances of unrealized expectations have come under my observation, that I have presumed to occupy a portion of this evening in considering the real advantages which may be looked for in a study of urine with the microscope.

Premising that such a range of power as is obtained by two objectives, an 8-10 and a 1-5 with two eye-pieces, an A and B, or a low medium power—that is from 80 to 400—will most usefully subserve our purposes, we may divide urine which is to be studied microscopically into *albuminous* and *non-albuminous.*

*Albuminous Urine.*—The urine with regard to which we may expect to derive most information, and in the study of which the microscope is indeed indispensable, is albuminous.

The first question to be determined with regard to albuminous urine is as to whether it contains casts of the uriniferous tubules. This question answered affirmatively, the general affection, Bright's disease, is recognized; the form of cast found to be most prevalent in connection with the quantity of albumen, and especially with the aid of the clinical history, enables us to determine the special form of Bright's disease, whether chronic or acute; and if the former, whether due to the smooth white kidney, the highly fatty organ, or the chronically contracted kidney, and even amyloid disease, with considerable certainty. And thus informed, matters of prognosis and treatment follow, the value of which no one can deny.

On the other hand, it is exceedingly seldom that the microscope enables us to decide the existence of cancerous from that of other destructive diseases of the kidney, as calculous pyelitis, the common purulent products being undistinguishable. Still less are we able to say, by means of the microscope alone, with regard to a limited number of pus or mucous corpuscles, that they are derived from the kidney rather than the bladder; at least, all attempts to this end are too speculative to be admitted to a place among the positive informations furnished by microscopic examination of urine.

Among the causes producing albuminous urine without the presence of casts, is the presence of pus; and although the same corpuscular element attends which is found in mucus, the albumen never accompanies mucus alone, while the distinctive characteristic mucin-threads developed on the addition of acetic acid to mucus furnishes the crucial information. This is apart from the physical characters of purulent urine, involved in the ready miscibility of the pus with the urine, its rapid subsidence and opacity as distinguished from the difficult miscibility of mucus, its transparency and slow deposition after mixture has been produced. Although albuminous urine, which is due to pressure upon the renal vein by a tumor or pregnant uterus, sometimes contains casts when the obstruction has produced actual congestion, this is comparatively rare, and the comfort which is derived by the practitioner from a knowledge that the albuminous urine of a pregnant woman does not contain casts, which the microscope alone can tell him, is unspeakable.

Urine which contains blood, from whatever source derived, is also albuminous. Except, however, when blood corpuscles are contained in casts of the uriniferous tubules, which indicates their undoubted renal origin, it can scarcely be claimed that the microscope is of much service in determining the exact source of the blood. It is rather the grosser characters, as the presence of coagula when blood is derived from the bladder, and the smoky hue of acid urine containing blood from the kidney, that gives us the desired information.

It is comparatively rare that albuminous urine results from affections of the bladder and prostate, except as the result of hemorrhage in malignant disease of the latter organs. In non-hemorrhagic malignant disease, attended by suppuration and rapid destruction of tissue, the urine may become impregnated with albumen, which will be explained by the presence of pus, and occasionally of fragments of tissue composed of the large multinuclear cell-masses, formerly considered so characteristic of cancer. In these cases, the almost inevitable though not indispensable accompaniment of vesical irritation will point to the bladder rather than the kidneys.

In the limited number of instances in which I have been permitted to examine the urine of patients who, as revealed by a *post-mortem* examination, suffered with cancer of the kidney, although albumen has been invariably present, I have never yet seen the cellular or other elements of cancer—nor, indeed, in cases of cancer of the bladder, though, in the latter, other observers have undoubtedly been more fortunate.

*Non-albuminous Urine.*—It must be admitted that the purely microscopic study of non-albuminous urine is not attended with so many advantages to the practitioner as that of albuminous. Still, there are numberless instances in which, at least, the clinical history of a case is not complete without a microscopic examination.

In no instance, perhaps, is the inexperienced person more frequently disappointed than in the examination of urine from cases of suspected calculi, both renal and vesical, but particularly the latter. Indeed, it may be laid down that, as a rule, except in uric acid lithiasis, the microscope alone rarely furnishes much information. To those who have had any experience, it is well known that in cases of phosphatic and oxalic lithiasis, the urine is commonly without any sediment, from the examination of which alone information can follow. With uric acid lithiasis, however, this is not the case, and very generally patients thus suffering have copious deposits of uric acid crystals. In the latter, therefore, we are able to make a positive diagnosis. The difficulty in the case of the phosphates is accounted for by these facts: The extreme solubility of the phosphates, and the dependence of their deposition upon the alkalinity of the urine; and in case of an exciting calculus, its power to excite, by decomposition of the surrounding organic matter, an alkalinity of the urine immediately around it, with consequent deposition of phosphates from such proximate urine, while the reaction of the great body of water continues acid. Occasionally, also, in the case of suspected oxalic calculus, information is derived by examination of urine from the constant presence of octohedral and dumb-bell crystals of oxalate of lime, especially if these be aggregated so as to form microscopic calculi of considerable size, as is often the case. If the symptoms of renal calculus are present, and such crystals be met repeatedly, we have good reason to believe that the calculus is of oxalic composition.—*Southern Medical Record.*

